# Golimumab improves work productivity in patients suffering from moderate to severe ulcerative colitis: results of a prospective study over 24 months

**DOI:** 10.1186/s12876-021-01747-z

**Published:** 2021-04-12

**Authors:** Niels Teich, Harald Grümmer, Eric Jörgensen, Thomas Liceni, Frank Holtkamp-Endemann, Tim Fischer, Susanne Hohenberger

**Affiliations:** 1 Internistische Gemeinschaftspraxis Für Verdauungs- Und Stoffwechselkrankheiten Leipzig Und Schkeuditz, Nordstraße 21, 04105 Leipzig, Sachsen Germany; 2 Praxis Für Innere Medizin/Gastroenterologie, Allee Nach Sanssouci 7, 14471 Potsdam, Germany; 3 Magen-Darm-Zentrum Remscheid, Rosenhügelerstraße 2, 42859 Remscheid, Germany; 4 MVZ Für Gastroenterologie Am Bayerischen Platz, Innsbrucker Straße 58, 10825 Berlin, Germany; 5 Gastroenterologische Gemeinschaftspraxis Am Germania-Campus, An der Germania Brauerei 6, 48159 Münster, Germany; 6 Medical Affairs, MSD Sharp & Dohme GmbH, Lindenplatz 1, 85540 Haar, Germany

**Keywords:** Golimumab, Ulcerative colitis, Work productivity and activity impairment (WPAI), Quality of life, Hospitalization, Health care resource utilization (HCRU)

## Abstract

**Background:**

Ulcerative colitis (UC) is a chronic inflammatory bowel disease with recurrent episodes of debilitating symptoms negatively affecting work productivity and health-related quality of life (HRQoL). The use of biologics in UC treatment improves work and HRQoL but prospective long-term data concerning the treatment with TNFα inhibitor golimumab in UC patients are still rare. Therefore, our study aimed to evaluate the change in work productivity, capacity for daily activities and HRQoL in UC patients treated with golimumab in Germany.

**Methods:**

Using the Work Productivity and Activity Impairment questionnaire, the change in work productivity and in capacity for daily activities after 3 months and over the whole observational period of 24 months were assessed (both primary endpoints). Disease-specific and health-related quality of life (QoL) were analyzed with the Inflammatory Bowel Disease Questionnaire (IBDQ), the Short-Form 12 Health Survey Questionnaire (SF-12), and the Partial Mayo Score (secondary endpoints). Further, disease-related hospitalization rates were assessed.

**Results:**

This prospective non-interventional study included 286 patients. Thereof, 212 patients were employed at baseline (modified intention to treat analysis set employed at baseline, mITTe). 61.3% of the mITTe patients had moderate and 17.0% had severe UC. Three months after initiation of golimumab therapy, total work productivity impairment (TWPI) score and activity impairment score improved significantly from baseline with a mean change of − 17.3% (p < 0.0001) and − 14.4% (p < 0.0001), respectively. Results persisted over 24 months (mean change TWPI score: − 24.5%, mean change activity impairment score: − 30.0%). Disease- and health-related QoL also improved significantly under golimumab treatment as indicated by increased IBDQ [mean change: 28.0 (SD: ± 36.1, month 3), 42.1 (SD: ± 39.5, month 24)] and SF-12 scores [PCS-12: 45.9 (SD: ± 8.5), MCS-12: 4.9 (SD: ± 10.6, month 3), PCS-12: 5.9 (SD: ± 9.0), MCS-12: 6.4 (SD: ± 11.1, month 24)]. Disease-related hospitalization rate decreased from 16.0% (BL) to 4.3% at month 24 and the mean number of missed working days due to UC decreased from 8.2 (SD: 17.6, BL) to 0.7 (SD: 2.1) after golimumab induction.

**Conclusions:**

Golimumab leads to notable long-term improvements in work productivity, daily activity, HRQoL, and disease-related hospitalization rates in patients with moderate to severe UC.

*Trial registration*: PEI (Paul-Ehrlich-Institute, Langen, Germany) Registration Nr: NIS#255 (https://www.pei.de/SharedDocs/awb/nis-0201-0300/0255.html)

## Background

Ulcerative colitis (UC) is a chronic inflammatory disorder of the gastrointestinal tract that typically begins in the second and third decades of life [1–3]. Mucosal inflammation extending proximally from the rectum and the development of extensive superficial ulceration are the key features [4]. Symptoms include recurrent flares of bloody diarrhea with numerous liquid bloody stools, fecal urgency, abdominal pain, incontinence, weight loss, and general malaise [3]. Moderate to severe ulcerative colitis highly impairs health-related quality of life (QoL) [5–8]. Additionally, a negative association between health-related QoL and unemployment, sick leave, and disability pension in patients with inflammatory bowel disease has been reported [9–12]. Since this represents a strenuous and challenging situation for the patients, rapid achievement and stable maintenance of clinical remission are the primary goals in UC therapy [13, 14].

Due to the early onset of UC and the associated high utilization of health services, UC causes significant socioeconomic burden [15, 16]. Since budget limitations and pressure on healthcare spending have been increased during the last years, data on indirect costs like productivity losses and health care resource utilization data have recently become extremely important in the German healthcare system [17]. Additionally, patient-reported outcomes and relevant additional benefit of new treatment options have come into focus of German sick funds to provide optimal medically and economically sensible healthcare services for affected patients [18].

Better understanding of the pathogenesis in UC has led to new treatment options such as tumor necrosis factor alpha inhibitors (anti-TNFα). The human monoclonal anti-TNFα antibody golimumab (Simponi^®^) is indicated for the treatment of moderately to severely active UC in adult patients who have had an inadequate response to conventional therapy, including corticosteroids and 6-mercaptopurine or azathioprine, or who are intolerant of or have medical contraindications to such therapies [19–21]. In moderate to severe Crohn´s disease, clinically meaningful improvement of work productivity and disease-specific QoL was found for anti-TNFα adalimumab [17, 22]. In the two *Active Ulcerative Colitis Trials 1* and *2* (ACT-1 and ACT-2, respectively) among others the relationships of clinical response and/or remission to another anti-TNFα, infliximab, with health-related QoL, employment, and productivity was assessed [23]. For patients in remission the improvements from baseline in productivity and both actual and fully productive hours worked per week were greater [24]. However, systematic data for golimumab in UC patients regarding work productivity and activity impairment as well as health-related QoL are still sparsely available in the literature. Therefore, the GO CUTE study was initiated to evaluate the changes in work productivity and Qol in UC patients treated with golimumab in Germany to characterize the benefit of this treatment option. Additionally, data on health economic aspects were collected to evaluate whether golimumab therapy may affect disease-related health care resource utilization.

## Methods

### Study design and study population

The GO CUTE study was a prospective, multicentre, open-label, non-interventional study conducted in 51 gastroenterological practices in Germany between March 2014 (first patient first visit) and July 2019 (last patient last visit). Biologic-naïve and biologic-experienced, golimumab naïve patients aged ≥ 18 years (inclusion criterion, IC 1), with UC diagnosed by a gastroenterologist (IC 2) who were suitable for golimumab therapy in accordance with the approved summary of product characteristics (SmPC) [19] and clinical standards (IC 3) and had given his/her signed consent form, were included in the study (IC 4). Patients with a contraindication according to the current Simponi^®^ (golimumab) SmPC (exclusion criterion, EC 1) [19], a previous treatment with golimumab (EC 2), with previous biologic treatment which was changed due to a serious adverse event (SAE), an opportunistic infection or hypersensitivity reaction (EC3), or/and a current participation in another clinical trial (with exception of register studies, EC 4) were not eligible for study participation.

Observational phase lasted 24 months starting with a baseline visit followed by 6 post-baseline visits in month 3, 6, 9, 12, 18, and 24 (end of observation). Treatment during observational phase was carried out in accordance with the current Simponi^®^ (golimumab) SmPC [19]. The decision to initiate golimumab therapy was made by the treating physician based on individual medical indication. According to the SmPC, golimumab was administered intravenously at a dose of 200 mg initially administered by subcutaneous injection in week 0, followed by 100 mg at week 2 and then 100 mg (if body weight ≥ 80 kg) or 50 mg (if body weight < 80 kg) every 4 weeks as maintenance therapy [19]. All patient-related data were recorded in pseudonymized form using an electronic case report form.

### Study endpoints and data collection

The primary endpoint of the study was to evaluate the changes in work productivity or activity impairment in month 3, 6, 9, 12, 18, and 24 (from baseline) compared to baseline in UC patients after induction of golimumab therapy using the work productivity and activity impairment questionnaire specified for UC (WPAI-UC V2.0) [25]. The WPAI (in general) represents the psychometrically best validated instrument for determining health-related work productivity [26, 27] and was proved in patients with UC in randomized, controlled trials as well as in non-interventional observational studies. All four WPAI scores (absenteeism, presenteeism, total work productivity impairment [TWPI], and daily activity) were assessed in the current study [28].

Secondary endpoints of the study were to assess the change in disease-related and health-related QoL determined by the Inflammatory Bowel Disease Questionnaire (IBDQ) and the 12-item Short-Form Health Survey Questionnaire (SF-12) as well as the change in disease activity measured by the Partial Mayo Score over 3, 6, 9, 12, 18, and 24 months. The IBDQ evaluates disease-related QoL and considers systemic and bowel symptoms as well as social function and emotional health by 32 items [29, 30]. The SF-12 comprises 12 questions on physical and mental status (physical component score [PCS-12] and mental component score [MCS-12]) to assess the overall state of health and evaluate the ability to engage in moderate activities [31]. The SF-12 is applicable regardless of the patients´ disease and age. The Partial Mayo Score is an instrument to measure disease activity without sigmoidoscopy by assessing stool frequency, rectal bleeding and physician’s global assessment (i.e. the three non-invasive components of the full Mayo score [32]). Each parameter ranges from 0–3, with a maximum total score of 9, where 0 to 1 = remission, 2 to 4 = mild disease, 5 to 6 = moderate disease, and 7 to 9 = severe disease (i.e. higher scores indicate worsening of the disease).

Assessment of changes in health care resource utilization, changes in the number of missed working days due to UC and changes in disease-related hospitalizations, each from baseline to month 12 as well as from month 12 to month 24, represented exploratory endpoints of the current study. Health care resource utilization assessment was deemed to provide an insight on UC impact on health care budgets [17]. Therefore, information concerning alternative treatment, rehabilitation, cure for UC or other reasons, ambulant, non-medical treatment, consultations due to UC, and UC-related sick leave (only for employed patients) were recorded. Using the Partial Mayo Score, the relationship of clinical baseline parameters and response to the therapy with health care resource utilization and work productivity can be assessed to identify patient groups with highest effects on health resources. Information about disease-related hospitalizations was derived from the physician’s own records, patient records in patient´s diary, and discharge summaries from the hospital where the patient was hospitalized. Disease-related was defined as any medical intervention with a direct, causal relation to UC, which aimed at the prevention of disease progression or prevention, reduction, or cure of symptoms/damage of UC or secondary diseases due to the underlying disease. Relationship was determined by the treating physician.

Furthermore, at each study visit, a complete physical examination was performed.

### Ethical considerations

This study was conducted in compliance with the principles of the Declaration of Helsinki. Prior to initiation of this non-interventional study at any site, the observational plan and the informed consent form were approved (reference number 13104) by the competent independent ethics committee of the Bavarian State Medical Association (Ethik-Kommission der Bayerischen Landesärztekammer, Munich/Germany). All study participants provided informed written consent prior to study enrolment. This non-interventional study was further registered at the Paul-Ehrlich-Institute, Langen, Germany (Registration Nr: NIS#255, https://www.pei.de/SharedDocs/awb/nis-0201-0300/0255.html).

### Statistical analysis

Statistical analyses were performed using SAS version 9.4. Categorical variables are presented as number and percentages. Quantitative variables are shown as mean with standard deviation (SD) or median with range. Differences or changes from baseline are indicated as mean change with SD or percentage change (%). Dependent sample t test was used for continuous variables if they were normally distributed. If the assumption of normal distribution was questionable, the Wilcoxon ranked sum test were performed to determine statistical significance. Differences were considered significant at p < 0.05. All enrolled patients who received at least one dose of golimumab, fulfilled all inclusion and exclusion criteria, had a baseline assessment and at least one documented additional visit, regardless of any protocol violation during the study and irrespective if he/she was still on golimumab treatment were included in the modified intention to treat (mITT) analysis set. Additionally, a mITTe subset was analyzed comprising of all patients of the mITT being full-time or employed at baseline.

## Results

### Patient disposition

**Fig. 1:**
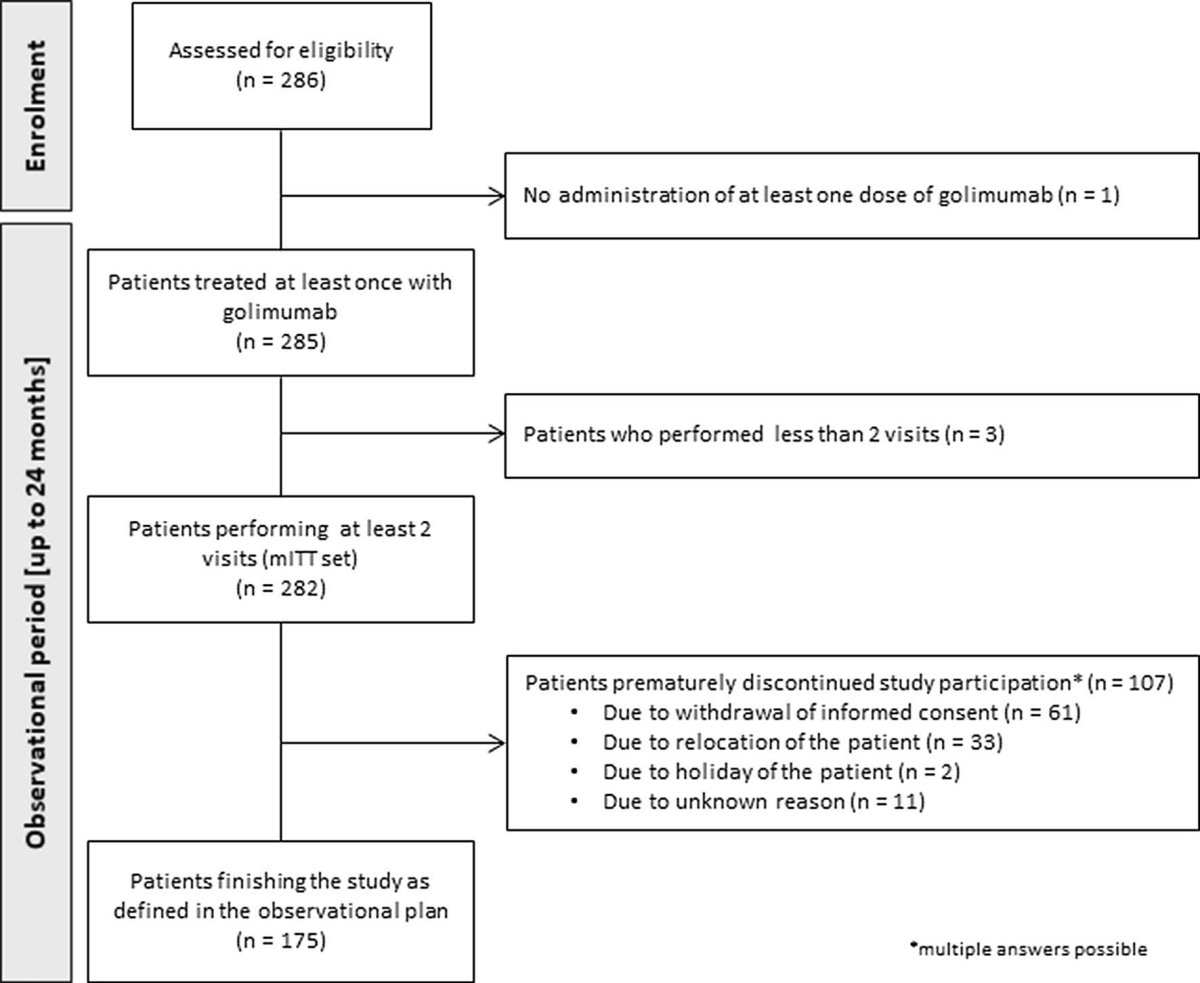
Patient flow diagram. n: number of patients, mITT: modified intention-to-treat analysis set

A total of 286 UC patients were enrolled in the study. One patient did not start treatment with golimumab; therefore, 285 patients remained in the study (Fig. 1). The mITT set consisted of 282 patients since three patients did not perform a second visit. As shown in Table 1, a total of 212 patients were employed at baseline and were evaluated for the primary endpoints of the study (mITTe). A total of 107 patients prematurely discontinued study participation, mostly due to withdrawal of informed consent (n = 61/107, Fig. 1). The gender balanced mITT population were aged from 18 to 95 years with a median age of 39.5 years (data not shown).

**Table 1:** Patient demographics and disease-specific properties at baseline

	mITT (n = 282)	mITTe (n = 212)
Male	134 (47.5)	102 (48.1)
Age mean (SD)	40.8 (± 13.9)	39.7 (± 12.0)
*Marital status*		
Single	116 (41.1)	88 (41.5)
Married/living together	156 (55.3)	117 (55.2)
Divorced	8 (2.8)	7 (3.3)
Widowed	2 (0.7)	-
*Stool frequency*		
Normal	33 (11.7)	22 (10.4)
1–2 stools more than normal	44 (15.6)	36 (17.0)
3–4 stools more than normal	94 (33.3)	71 (33.5)
≥ 5 stools more than normal	111 (39.4)	83 (39.2)
*Rectal bleeding*		
No blood seen	95 (33.7)	68 (32.1)
Streaks of blood with stool less than half the time	80 (28.4)	54 (25.5)
Obvious blood with stool most of the time	82 (29.1)	70 (33.0)
Blood alone passes	25 (8.9)	20 (9.4)
*Physician global assessment*		
Normal	8 (2.8)	7 (3.3)
Mild disease	54 (19.1)	39 (18.4)
Moderate disease	172 (61.0)	130 (61.3)
Severe disease	48 (17.0)	36 (17.0)
*Alcohol consumption (actual)*		
No	143 (50.7%)	115 (54.2)
Yes	139 (49.3%)	97 (45.8)
*Frequency of alcohol consumption (n = 139)*		
Occasional	117 (81.8)	95 (82.6)
Once a week	12 (8.4)	11 (9.6)
Several times a week	9 (6.3)	5 (4.3)
Daily	5 (3.5)	4 (3.5)
*Nicotine consumption*		
No	254 (90.1)	189 (89.2)
Yes	28 (9.9)	23 (10.8)
*Abuse of other substances*		
No	281 (99.6)	211 (95.5)
Yes (Cannabis)	1 (0.4)	1 (0.5)

Data are shown as n (%), if not stated otherwise. mITT: modified intention to treat analysis set, mITTe: all patients of the mITT who had been employed at baseline

UC was the most frequently stated reason for full reduction of work (n = 8/12) or incapacity to work (n = 11/11) but not for working in part-time (20.0%, n = 8/40, Table 2).

**Table 2:** Occupational status at baseline

		mITT (n = 282)	mITTe (n = 212)
*Occupational status*			
Employed (full time)		172 (61.0)	172 (81.1)
Employed (part time)		40 (14.2)	40 (18.9)
Retired		12 (4.3)	n. a
Full reduced		12 (4.3)	n. a
Incapable of working		11 (3.9)	n. a
*Part time employment due to (n = 44)*			
Ulcerative colitis		8 (20.0)	8 (20.0)
Other reason		32 (80.0)	32 (80.0)
*Full reduced due to (n = 12)*			
Ulcerative colitis		8 (66.7)	n. a
Other reason		4 (33.3)	n. a
*Reason for incapable of working (n = 11)*			
Ulcerative colitis		11 (100.0)	n. a

Data are shown as n (%). mITT: modified intention to treat analysis set, mITTe: all patients of the mITT who had been employed at baseline

Nearby half of the patients (45.0%, n = 127/282) reported at least one concomitant disease at BL (Table 3).

**Table 3:** Concomitant diseases, extraintestinal manifestations and concomitant medication at baseline

	mITT (n = 282)
Patients with concomitant disease	127 (45.0)
Patients with EIMs of UC^a^	31 (11.0)
Joint	24 (8.5)
Other	13 (4.6)
Patients extraintestinal concomitant disease associated with UC^a^	5 (1.8)
Gall stone	3 (1.1)
Thromboembolic complications	2 (0.7)
Kidney stone	1 (0.4)
*Common other concomitant diseases*^a^	
Hypertension	28 (10.2)
Depression	9 (3.2)
Asthma	7 (2.5)
Osteoporosis	7 (2.5)
Other	23 (8.2)
*Concomitant medication*^a^	
No	85 (30.1)
Yes	197 (69.9)
Aminosylicylates	117 (41.5)
Corticosteroids	68 (24.1)
Immunosuppressives	39 (13.8)
Analgesics	15 (5.3)
Loperamid	7 (2.5)
Other^b^	86 (30.5)

Data are shown as n (%)

^a^Multiple answers were possible. mITT: modified intention to treat analysis set. EIM: extraintestinal manifestations

^b^Other drugs mainly comprised antihypertensive drugs, antidiuretic agents, antidepressants, supplements, antidiabetics, oral contraceptives and non-steroidal anti-inflammatory drugs

For 11.0% of the patients (n = 31/282 patients) extraintestinal manifestations of UC were documented. The majority of patients (69.9%, n = 197/282 patients) received concomitant medication at baseline, including aminosalicylates (41.5%, n = 117/282) and corticosteroids (24.1%, n = 68/282, Table 3). Additional details concerning patient demographics and disease characteristics, occupational status as well as concomitant diseases and concomitant medications are shown in Tables 1, 2, and 3, respectively.

### Work productivity and activity impairment in UC patients (mITTe set)

**Fig. 2:**
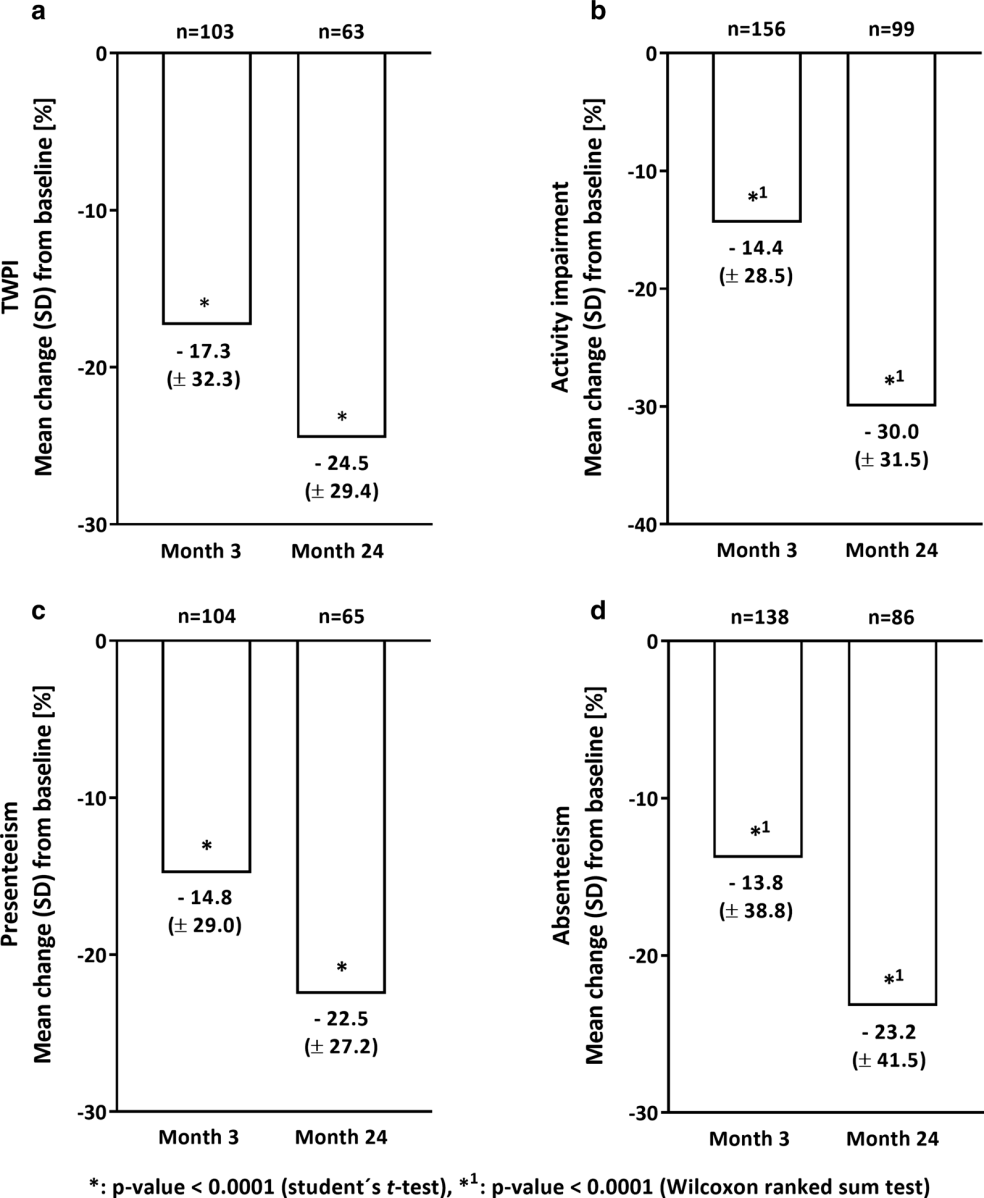
Work productivity and activity impairment in UC patients (mITTe set). The mean change in total work productivity impairment (TWPI, **a**) and activity impairment (**b**) is shown for month 3 and for month 24 (both primary endpoints). For both impairments a statistically significant improvement was observed at month 3 and month 24, respectively. For absenteeism (**c**) as well as presenteeism (**d**), similar outcomes were reported. **a**–**d** Analyses were performed for the mITT analysis set employed at BL (mITTe set). Data were shown as mean change (SD) from baseline in %. n: number of patients. BL: baseline. SD: standard deviation

After 3 months of onset of golimumab treatment a statistically significant reduction in all WPAI-UC scores compared to baseline was observed (p value < 0.0001, t test or Wilcoxon ranked sum test, Fig. 2a–d). For total work productivity impairment, a mean change of − 17.3% (SD: ± 32.3) was detected (Fig. 2a). At the end of the study, the mean value in the total work productivity impairment subscore decreased from 49.4% (SD: ± 27.8, baseline) to 23.4% (SD: ± 23.8, month 24). This corresponded to a mean change of − 24.5% (SD ± 29.4, Fig. 2a). Similar results were observed for the activity impairment score with a reduction of − 14.4% (mean change, SD: ± 28.5, month 3) and − 30.0% (mean change, SD: ± 31.5, month 24) from baseline to the respective timepoints (Fig. 2b). For the subscore absenteeism, a mean change from baseline of − 13.8% (SD: ± 38.8) at month 3 and a mean change of − 23.2% (SD: ± 41.5) at month 24, respectively, was observed (Fig. 2d). The mean change from baseline of the presenteeism subscore was − 14.8% (SD: ± 29.0) after 3 months and − 22.5% (SD: ± 27.2) at month 24, respectively.

### Disease- and health-related QoL in UC patients (mITT set)

**Fig. 3:**
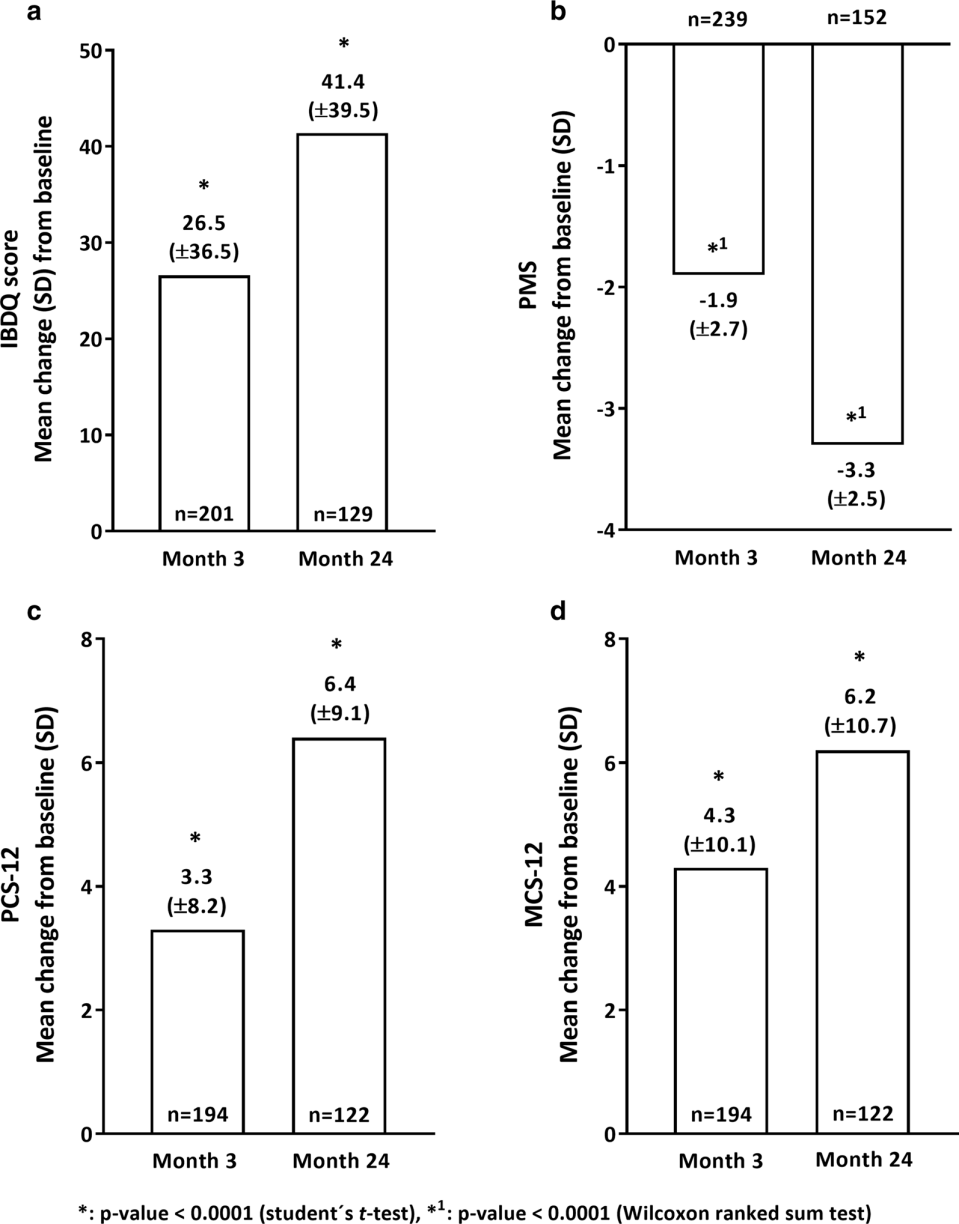
Disease- and health-related quality of life and disease severity in UC patients (mITT). The mean change of the total Inflammatory Bowel Disease Questionnaire score is presented in **a** revealing a statistically significant change in month 3 and month 24 in comparison to BL. A statistically significant decrease in disease severity was observed in month 3 as well as in month 24 after onset of golimumab treatment as shown in **b**. Health-related quality of life measured by SF-12 (**c** [PCS-12], **d** [MCS-12]) was also improved in UC patients at both time points when compared to BL data. Analyses were done for the mITT analysis set and p values were generated using the paired t test (*) or Wilcoxon ranked sum test (*^1^). n: number of patients. BL: baseline. SD: standard deviation. IBDQ: inflammatory bowel disease questionnaire. PMS: partial Mayo score. SF-12: 12-item short form health survey. PCS-12: physical condition score of the SF-12. MCS-12: mental condition score of the SF-12

After onset of golimumab treatment, a significant improvement of disease-related QoL measured by the Inflammatory Bowel Disease Questionnaire was observed (p value < 0.0001, t test, Fig. 3a). After 3 months, mean change from baseline in total IBDQ score was 26.5 points (SD: ± 36.5, Fig. 3a). At the end of the 24 months study, mean change in Inflammatory Bowel Disease Questionnaire score amounted to 41.4 (SD: ± 39.5) points. Health-related QoL, evaluated by the 12-item Short-Form Health Survey Questionnaire, was also markedly improved in UC patients since the majority of patients (n = 194) had a significant increase in both subscores of the 12-item Short-Form Health Survey Questionnaire after 3 months [PCS-12: mean change from baseline of 3.3 (SD: ± 8.2) points, MCS-12: mean increase of 4.3 (SD: ± 10.1) points] as well as after 24 months [PCS-12: + 6.4 (SD: ± 9.1) points, MCS-12: + 6.2 (SD: ± 10.7) points, for both subscores: p value < 0.0001, t test, Fig. 3c, d].

### Disease severity and disease-related hospitalizations (mITT set)

Physicians assessed disease severity by the Partial Mayo Score. Three months after baseline, the Partial Mayo Score decreased notably with a mean change of − 1.9 (SD: ± 2.7) points (mITT set, p value < 0.0001, Wilcoxon ranked sum test, Fig. 3b). A significant reduction in Partial Mayo Score was also observed after 24 months, with a mean change from baseline of − 3.3 (SD: ± 2.5) points in two third of the patients (n = 152, mITT set, p value < 0.0001, Wilcoxon ranked sum test, Fig. 3b).

**Fig. 4:**
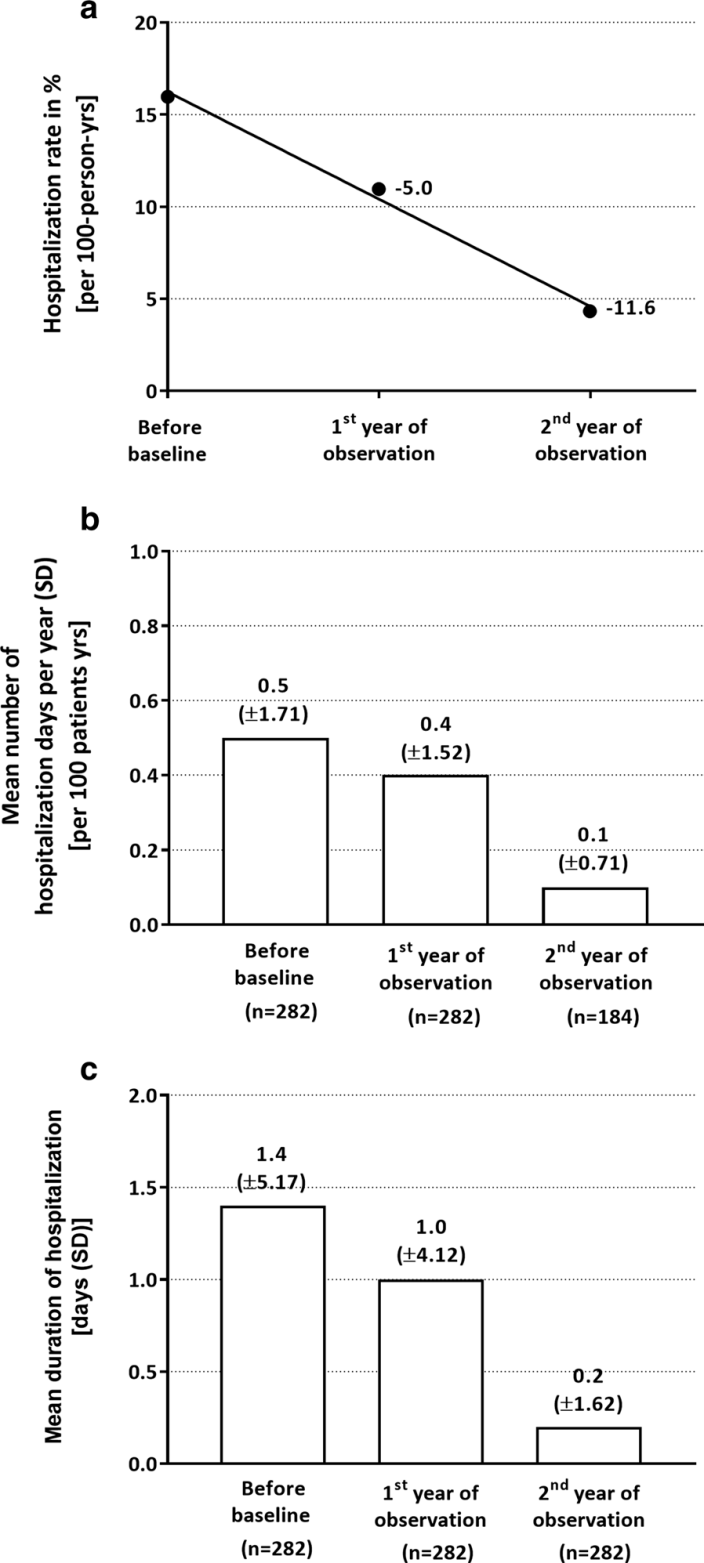
Disease-related hospitalizaton rate. The hospitalization rate of the mITT set is shown in **a** for the year before baseline (BL) visit and for the 1st (month 12) and 2nd year (month 24) of observation. **b** displayed the mean number of hospitalization days per 100 patient years with SD before BL and during the observational period. The mean duration of hospitalization in days is shown in **c**. Linear regression line was calculated using Graph Pad Prism 7.05. n: number of patients. Yrs: years

During the observational period, the disease-related hospitalization rate (per 100-person-years) decreased from 16.0% to 11.0% (after 1st year of observation, mean change of − 5.0 [95%CI: − 8.73, − 1.26]) and to 4.3% (after 2nd year of observation, mean change of − 11.6 [95%CI: − 13.55, − 9.71], Fig. 4a). At baseline (based on the year before), the mean number of disease-related hospitalization days was 0.5 (SD: ± 1.71) per 100 patient years (Fig. 4b). After 1st year of observation, just a minor decline in the mean number of disease-related hospitalization days was observed [0.4 days (SD: ± 1.52) per 100 patient years] whereas after the 2nd year of observation, the mean number of disease-related hospitalization days was notably declined to 0.1 days (SD: ± 0.71) per 100 patient years (Fig. 4b). Additively, a reduction in the mean duration of hospitalization was observed during observational period [from 1.4 days (SD: ± 5.17) (year before baseline) to 1.0 days (SD: ± 4.12) after 1st year of observation and to 0.2 days (SD: ± 1.62) after 2nd year of observation, Fig. 4c].

### Health care resource utilization and missed working time

**Fig. 5:**
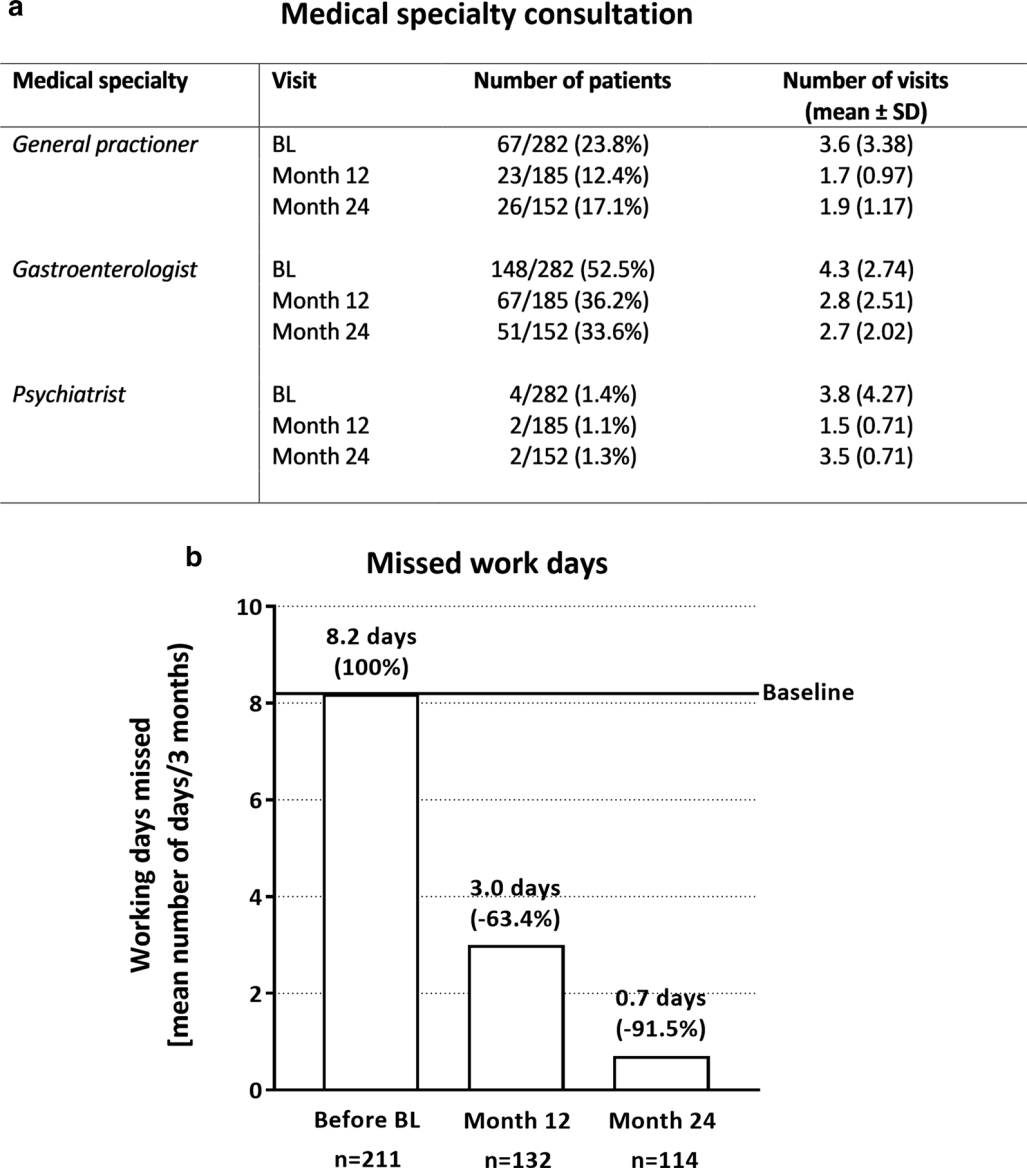
Disease-related health care resource utilization und working time missed. Disease-related health care resource utilization (HCRU) were assessed among other items by the number of medical specialty consultations in regard to UC in the mITT analysis set. An overview is given in **a**. Additionally, the missed working time in the 3 months before BL and after 3 and 24 months after start of golimumab treatment in the mITTe analysis set is shown in **b** indicated as mean number of days/3 months. A marked decrease in the number of missed working days was observed after onset of golimumab treatment. BL: baseline. n: number of patients

Health care resource utilization assessment revealed that the number of patients seeking general practitioners [baseline: 23.8% of the patients (n = 67/282); month 24: 17.1% of the patients (n = 26/152)] or gastroenterologists [baseline: 52.5% of the patients (n = 148/282); month 24: 33.6% of the patients (51/152)] for medical advice declined after onset of golimumab treatment (Fig. 5a). Additionally, a slight reduction in the mean number of visits were observed [general practitioners: from 3.6 (SD: ± 3.38, baseline) to 1.9 (SD: ± 1.17, month 24), gastroenterologists: from 4.3 (SD: ± 2.74, baseline) to 2.7 (SD: ± 2.02, month 24), Fig. 5a]. The number of patients utilizing alternative treatment [baseline: 3.6% (n = 10/282) of the patients, month 24: 1.3% (n = 2/152) of the patients] or ambulatory treatment [baseline: 3.9% (n = 11/282) of the patients, month 24: 2.0% (n = 3/152) patients], was also reduced after golimumab treatment (data not shown). Three months before baseline, the mean number of working days missed was 8.2 days/3 months. After 1st year of observation, this number declined to 3.0 days/3 months. At the end of the observational phase, solely 0.7 working days/3 months were missed by the patients (Fig. 5b).

## Discussion

The GO CUTE study was a prospective, multicenter study in Germany, demonstrating that golimumab treatment markedly improved work productivity and ability to perform daily activities in UC patients 3 months after induction of golimumab. These benefits were already shown in a 12-months interim analysis [33] but persisted over the whole observational period of 24 months. Furthermore, golimumab treatment led to a reduction of missed working days and to a marked improvement of health- and disease-related QoL shown by increase scores of Inflammatory Bowel Disease Questionnaire and 12-item Short-Form Health Survey Questionnaire. Additionally, a decline of the disease-related hospitalization rates after 1st and 2nd year of observation with shorter residence times in hospital were observed after start of golimumab treatment. Evaluation of UC-related health care resource utilization showed that the number of patients with medical specialty consultations markedly decreased and less ambulatory treatments have been utilized by the study population during observational period.

UC patients with active disease have severely impaired work life since they are faced with lower ability to concentrate, reduced working speed and minor work productivity additionally to gastrointestinal symptoms leading to a notable reduction in health-related QoL [9, 11]. Beside the affected patients, UC-dependent work impairment and incapacity cause an increase of direct and indirect healthcare costs [15, 16]. Therefore, an adequate maintenance of disease is important for the patients and the healthcare system. Our results showed that golimumab positively affects the work productivity in UC patients and reduces missed working days. These findings support the results of studies such as the *Active Ulcerative Colitis Trials 1* and *2 *which investigated the effect of biologic treatment on UC [24]. Those trials demonstrated that UC patients who achieved clinical remission or response were able to increase their hours per week (i.e., hours actually worked) as well as their work productivity. Further, they improved their disease- and health-related QoL as observed by increased Inflammatory Bowel Disease Questionnaire and 36-item Short Form Health survey scores. In the GO CUTE study, most of the patients also achieved clinical remission and an improved QoL, both representing well-accepted therapeutic goals in UC treatment [14, 34]. Consequently, golimumab represents, such as other anti-TNFs [17, 35], an effective therapeutic option to improve work life conditions and patients´ well-being in those people suffering from moderate to severe UC.

UC represents a cost-intensive disease [36], therefore health insurance providers are interested in medical and economical effects of new treatment options. Our results revealed that beside an improved work life and an enhanced QoL, golimumab decreased the number of disease-related hospitalizations in treated patients over 24 months. A reduced hospitalization rate together with reduced medical specialist consultations after golimumab induction as observed in the current study, appear to lower costs of health care resource utilization. Several studies reported that there is a shift from UC-related hospitalization costs due to costs caused by biologics-based treatment in patients with UC [37]. However, patients with reduced number of hospitalizations also reported an improved disease- and health-related QoL [38] and consequently might use healthcare services less often than patients with active disease. Our study supports such observations since on the one hand, most patients achieved an enhanced QoL and on the other hand, health care resource utilization decreased during observational phase. Those findings underline the necessity to consider all aspects of a treatment option to ensure a reasonable pharmacoeconomic evaluation by health policy makers to support the medically most appropriate and resource-conserving treatment that effectively normalizes QoL in UC patients.

Remarkable, large number of patients had withdrawn informed consent during observational phase. Several studies reported a higher rate of treatment failure in patients treated with golimumab than in patients receiving adalimumab, infliximab or a biosimilar to infliximab often associated with a treatment switch [39]. Such a switch could be a reason for patients to withdraw their informed consent, but patients are not obliged to explain their motives, thus only speculations will be possible. Due to a high rate of withdrawn informed consents, the number of patients with adequate treatment response could be overrepresented in the current study. However, since patients were selected by the treating physician in accordance to the current SmPC for golimumab treatment independently of study inclusion, we think, the study represent the real-world situation in Germany and therefore, results are reliable and meaningful.

In summary, the GO CUTE study illustrated that golimumab is an effective treatment in patients suffering from moderate to severe UC in this real-world outpatient setting. Golimumab treatment allowed sustained improvement in WPAI and disease- and health-related QoL. Further, after golimumab induction a notable reduction of disease-related hospitalizations and health care resource utilization is observable which also improved disease-related QoL.

## Conclusions

Golimumab treatment improves notably patients´ work productivity and daily activity as well as disease- and health-related quality of life over 24 months after golimumab induction.

## Data Availability

The datasets used and/or analysed during the current study are available from the corresponding author on reasonable request.

## Abbreviations

Anti-TNFα: Anti tumor necrosis factor alpha
BL: Baseline
EC: Exclusion criterion
HCRU: Health care resource utilization
HRQoL: Health-related quality of life
IBDQ: Inflammatory bowel disease questionnaire
IC: Inclusion criterion
MCS-12: Mental component score of the SF-12
mITT: Modified intention to treat analysis set
mITTe: Modified intention to treat analysis set (employed at baseline)
PCS-12: Physical component score of the SF-12
PMS: Partial Mayo score
QoL: Quality of life
SD: Standard deviation
SF-12: 12-Item short-form health survey questionnaire
SmPC: Summary of product characteristics
TNFα: Tumor necrosis factor alpha
TWPI: Total work productivity impairment
UC: Ulcerative colitis
WPAI: Work productivity and activity impairment questionnaire
WPAI-UC: Work productivity and activity impairment questionnaire – ulcerative colitis
